# Food Insecurity and Rural Child and Family Functioning

**DOI:** 10.1001/jamanetworkopen.2025.30691

**Published:** 2025-09-05

**Authors:** Merelise R. Ametti, Hannah E. Frering, Kierian Huang, Katya Marsh, Robert R. Althoff

**Affiliations:** 1 Department of Psychiatry, Larner College of Medicine at the University of Vermont, Burlington; 2 Center for Clinical and Translational Science, MaineHealth Institute for Research, Scarborough; 3 Institute for Neuroscience, University of Nevada, Reno; 4 College of Education and Social Services, University of Vermont, Burlington

## Abstract

**Importance:**

In the US, children in food-insecure households are at risk for adverse psychological outcomes despite being shielded from hunger and malnutrition by their caregivers and school- and community-based programs. Parenting stress may be an important mechanism through which food insecurity is associated with negative outcomes for child mental health.

**Objective:**

To investigate associations of household food insecurity with child mental health, parenting stress, and family functioning.

**Design, Setting, and Participants:**

This cross-sectional study used a daily-diary design in a community-based sample to examine associations between daily fluctuations in food insecurity and child and family functioning. The study was conducted remotely during the COVID-19 pandemic in the rural (defined by the Health Resource Services Administration) Northeastern US. Caregivers of school-aged children were enrolled during the 2021 school year and completed a baseline visit followed by mobile surveys for 30 days. Participants were adult, legal caregivers of children ages 6 to 12 years who experienced food insecurity within the past month. Participants were required to have English proficiency and access to a device with internet and texting capabilities. Prospective participants completed an online questionnaire to determine eligibility. A total of 553 respondents were screened, of which 327 respondents (59.1%) met all inclusion criteria. The most common reason for ineligibility was not having a child in the study age range. Data analyses were performed between May 2022 and April 2023.

**Exposure:**

Household food insecurity.

**Main Outcomes and Measures:**

The primary outcomes were caregiver hunger, negative affect, executive functioning, and parent-child interactions (measured via a daily self-report survey) and child mental health problems (measured via the Child Behavior Checklist [CBCL]).

**Results:**

Among 61 caregivers (mean [SD] age, 36.1 [5.9] years; 51 women [83.6%]; 2 American Indian or Alaska Native [3.3%], 1 Black [1.6%], and 55 non-Hispanic White [90.2%]), greater day-to-day fluctuations in household and child food insecurity were associated with more severe child internalizing problems (CBCL Internalizing Problems scale: β = 0.40; *P* = .003 for household and β = 0.49; *P* < .001 for child food insecurity) and total mental health problems (CBCL Total Problems scale: β = 0.34; *P* = .01 for household and β = 0.35; *P* = .01 for child food insecurity). Additionally, the daily association between food insecurity and parent-child conflict was fully mediated via 2 sequential pathways of caregiver hunger and negative affect (*b* = .02; *P* = .001) and caregiver hunger and attention and impulse control (*b* = 0.01; *P* = .04). This model explained 17% of variability in daily parent-child conflict (*R*^2^ = 0.17).

**Conclusions and Relevance:**

This study’s findings suggest that caregiver stress and household instability may be key mechanisms by which food insecurity is negatively associated with child mental health.

## Key Points

**Question:**

Is food insecurity associated with caregiver well-being, family relationships, and child mental health?

**Findings:**

In this cross-sectional study of 61 child caregivers in rural food-insecure households conducted during the COVID-19 pandemic, greater day-to-day fluctuations in food security were associated with child mental health problems. Daily levels of household food insecurity were positively associated with caregiver hunger, negative affect, and parent-child conflict and negatively associated with caregiver perceived effectiveness and attention and impulse regulation.

**Meaning:**

These findings suggest that household food insecurity may be indirectly associated with adverse outcomes for child mental health through household chaos and caregiver stress.

## Teaser

This cross-sectional study investigates the association of household and child food insecurity with child mental health, parenting stress, and family functioning.

## Introduction

Food insecurity is a household-level economic and social condition in which access to adequate, nutritious, and safe food is limited or uncertain.^1^ In the US, experiences of food insecurity are prevalent and heterogenous, affecting 17% of households with children and ranging in severity from worries about food sufficiency (“marginal food security”) to reliance on unhealthy, inexpensive foods and cutting down or skipping meals (“low food security”) to going a full day or more without food (“very low food security”). Members of racial and ethnic minority groups, single mothers, older adults, and individuals with disabilities, mental illness, and chronic medical conditions are disproportionately impacted by food insecurity.^2,3,4,5^ Rural households face additional barriers to food security given that long distances to supermarkets often necessitate reliance on convenience stores and gas stations, where healthy, quality foods tend to be less available and considerably more expensive.^6,7,8^ Notably, the COVID-19 pandemic also contributed to an increase in rates of food insecurity, including many newly food-insecure families.^1,2,3^

Children in food-insecure households are usually conferred protection against hunger and malnutrition through participation in programs, such as the National School Lunch and Breakfast programs, and caregivers who sacrifice their own intake.^9,10,11,12^ Nonetheless, research suggests that children are often aware and worried about their families’ food situation and covertly engage in strategies to obtain or conserve food.^13^ Moreover, children in food-insecure households exhibit adverse developmental outcomes and increased mental health problems, including hyperactivity, disruptive behaviors, language delays, anxiety, depression, suicidality, substance use, and absenteeism.^14,15,16,17,18,19,20^

To mitigate these disparities among food-insecure households, there is a critical need to better understand the mechanisms through which food insecurity is associated with negative child mental health outcomes. Caregiver mental health and parenting are potential pathways.^21,22,23,24^ In addition to experiencing the most severe dietary disturbances within their households, caregivers at all levels of food insecurity are at risk of increased anxiety, depression, irritability, guilt, and isolation, as well as more difficulties with attention and executive functioning.^22,24,25^ This parenting stress is often associated with harsher, less emotionally responsive parenting behaviors,^26^ a well-established risk factor associated with child mental health problems.

Traditional measures of food insecurity, which broadly assess household experiences over an extended time (eg, 12 months), have presented a barrier to interrogating these mechanisms by which food insecurity may be associated with impaired family functioning.^27^ However, more recently, daily-diary designs have been used to study food insecurity,^27,28^ allowing for dynamic, temporally specific information about each household’s experience of food insecurity and its associated outcomes.^27,29^ The goal of the this study was to use this novel approach to describe in detail the experiences and functioning of rural children and caregivers experiencing food insecurity during the COVID-19 pandemic.

## Methods

All procedures for this cross-sectional study were approved by the University of Vermont Institutional Review Board. Due to COVID-19 restrictions, participants provided electronic informed consent via Research Electronic Data Capture (REDCap).^30,31^ This study is reported following the Strengthening the Reporting of Observational Studies in Epidemiology (STROBE) reporting guideline.

### Participants and Recruitment

Participants were 61 caregivers of school-aged children living in rural counties in the Northeastern US who experienced food insecurity. They were recruited through social media advertisements and flyers and screened by staff to ensure that they met all eligibility criteria (Box).

Box. **Eligibility Criteria**

**Inclusion Criteria**

1. Aged ≥18 y
2. Legal guardianship of a child aged 6-12 y with whom they live ≥75% of the time
3. Worries about food insufficiency or actual food insufficiency within the past mo
4. Residence in a county designated as rural by the HRSA

**Exclusion Criteria**

1. Unable to read or write in English (<fifth-grade level)
2. Lack of access to technology with text messaging and internet capabilities
3. Known caregiver diagnosis of intellectual disability
4. Caregiver incarceration

Abbreviation: HRSA, Health Resources and Services Administration.

### Procedures

Study participation involved a baseline study visit and 1 month of daily surveys completed via text message. Participants were continuously enrolled from March to June and September to December 2021. Data collection was paused during the kindergarten through grade 12 summer vacation to avoid potential confounds related to changes in family routines and services.

#### Baseline Data Collection

Baseline study visits were conducted remotely via videoconference or telephone. After providing informed consent, participants completed online questionnaires and a computerized cognitive assessment, which were intended to capture global characteristics of the child, caregiver, and family’s functioning. Participants were then registered to receive daily surveys via Mosio, an automated text messaging program (Mosio, Inc).

#### Mobile Data Collection

Daily mobile surveys began the day after the baseline visit and continued for 30 days, assessing household food insecurity, caregiver negative affect, hunger, executive function, and parent-child interactions. Surveys were distributed in the evenings at times tailored to family schedules and remained open until the following morning to accommodate night shifts. Surveys required approximately 10 minutes per day. Participants were compensated following an escalating payment schedule, starting at $2 per survey during the first week and increasing by $1 per week up to $6 per survey.

### Measures

#### Baseline Assessment

Child mental health symptoms and academic performance were measured using the Child Behavior Checklist (CBCL) via 120 problem items rated on a 3-point scale.^32^ Items yield empirically derived subscales related to different psychological symptoms and competence in several areas of functioning, which are normed by age, gender, culture, and informant. *T-*scores (mean [SD] score, 50 [10]) on Total Problems, Internalizing Problems, Externalizing Problems, Attention Problems, and Academic Performance subscales were used.^32^

Additional covariates collected at baseline included caregiver self-reported demographic information (eg, age, gender, and race and ethnicity), IQ (Raven Advanced Progressive Matrices),^33^ mental health symptoms (Adult Self-Report),^34^ and physical functioning (Short Form Health Survey)^35^ (eAppendix 1 in Supplement 1). Race and ethnicity were collected to assess the local and national representativeness of the sample. Caregivers self-reported all applicable identities from the following categories: American Indian or Alaska Native; Asian; Black or African American; Hispanic, Latino, or Spanish origin; Middle Eastern or North African; Native Hawaiian or Other Pacific Islander; White; and other not listed. Options for race and ethnicity were presented as a single question that allowed participants to endorse all applicable categories.

#### Daily Questionnaire

The daily survey consisted of 42 multiple-choice questions, which included 5 items adapted from the US Department of Agriculture Household Food Security Survey.^36^ We used 3 questions assessing food insecurity at the household level that were derived from a previous study of daily food insecurity^28^ and 2 additional questions that captured caregiver perceptions of the direct effects of food insecurity on children in the household (Table 1). Total scores ranged from 0 to 7 for household food insecurity and 0 to 3 for child food insecurity.

**Table 1.** Daily Food Insecurity Questions

Item	Response options
1. Today, did you worry whether food would run out before you got money to buy more?	0 = No; 1 = yes
2. Today, how much did you feel that you could not afford to eat balanced meals?	0 = Not at all; 1 = somewhat; 2 = a lot
3. Today, did you eat less food than you felt you should because there wasn’t enough money to buy food?	0 = No; 1 = yes
4. Today, how much did you feel you could not afford to feed your children balanced meals?	0 = Not at all; 1 = somewhat; 2 = a lot
5. Today, did your children eat less than you felt they should because there wasn’t enough money to buy food?	0 = No; 1 = yes

In addition, 5 items modified from the Positive and Negative Affect Schedule^37^ assessed caregiver daily negative affect, 8 items modified from the Attentional Function Index^38^ measured caregiver perceived level of daily cognitive dysfunction and its effects on activities, and 8 items from the Block Child-Rearing Practices Report^39^ were used to describe daily parent-child interactions. Caregivers were also asked about daily levels of hunger and the amount of time spent with their child.

### Statistical Analysis

Data analysis was conducted using MPlus software version 8.5 (Muthén and Muthén).^40^ Data analyses were performed between May 2022 and April 2023.

#### Outcomes Among Children

The sum of each participants’ responses on daily food insecurity items were used to compute intraindividual means (iMeans) and SDs (iSDs), representing each households’ mean level of food insecurity over the month and their mean deviation from their mean level of food insecurity each day, respectively (eAppendix 2 in Supplement 1). The iMean and iSD for household food insecurity were included simultaneously as independent variables in each model examining associations with child mental health symptoms and academic performance. Separate models were performed for each outcome. Analyses were repeated using the iMean and iSD for items regarding children’s direct experiences of food insecurity as independent variables.

#### Outcomes Among Caregivers

The association between household food insecurity and caregiver daily functioning was examined using a multilevel structural equation modeling (MSEM) framework, which decomposes variability in observed variables into within-person (level 1) and between-person (level 2) variances and covariances, accounting for measurement error.^41^ Specifically, at level 1, a sequential mediation model was specified whereby daily household food insecurity was associated with same-day parenting behavior directly and indirectly via caregiver hunger, affect, and executive functioning. At level 2, an analogous model was specified testing associations between the mean of each construct across time points. Analyses used 1830 person-days (61 participants × 30 days). Before structural models were conducted, psychometric properties of daily items were analyzed by computing intraclass correlations and multilevel confirmatory factor analysis models to identify latent constructs. MSEM models included random intercepts and fixed slopes. To account for potential temporal trends, the number of days since the start of the study and since Supplemental Nutrition Assistance Program (SNAP) benefit transfer were included as exogenous variables. Models were first analyzed without covariates; then, caregiver age, gender, IQ, mental health problems, physical functioning, SNAP participation, daily hunger, and time spent with children were added in a stepwise fashion to examine the contributions of each to model fit. We appraised model fit using commonly accepted fit indices for MSEM: root mean square error of approximation (RMSEA; values ≤.08 = acceptable; values ≤.05 = good), comparative fit index (CFI; values ≥.90 = acceptable; values ≥.95 = good), and standardized root mean square residual (SRMR; values ≤.10 = acceptable; values ≤.08 = good).

## Results

### Sample Characteristics

A total of 553 potential candidates were screened for eligibility, of which 327 candidates (59.1%) met all inclusion criteria. Of these eligible candidates, 266 individuals did not respond to outreach. The most common reason for exclusion was not having a child ages 6 to 12 years. Fewer than 6% of candidates were excluded due to lacking smartphone or internet access (13 candidates [5.8%]), consistent with national data showing high rates of smartphone and other device ownership among lower-income and rural individuals.^42^ There were no differences between respondents who enrolled and did not enroll with regard to age, severity of food insecurity, number of children in the household, or participation in SNAP.

A total of 61 caregivers were enrolled in the study (mean [SD] age, 36.1 [5.9] years; 51 women [83.6%]; 2 identified as American Indian or Alaska Native [3.3%], 1 identified as Black or African American [1.6%], and 55 identified as non-Hispanic White [90.2%]) (Table 2). Race and ethnicity demographics were consistent with the composition of the catchment area.^42^ Nearly half of caregivers were single parents (26 caregivers [42.6%]). During the past year, 32 participants (52.5%) reported their food security status as “very low,” whereas 26 participants (42.6%) and 2 participants (3.3%) reported “low” and “marginal” food security, respectively. In addition, 32 households in the sample (52.5%) received SNAP benefits. Participants receiving SNAP benefits were significantly more likely to be single parents, have less than a 4-year college education, and live in a household with an income of less than $30 000 per year. Caregivers reported on 61 children (36 male [59.0%]; mean [SD; range] age, 9.1 [1.9; 6-12] years).

**Table 2.** Caregiver and Household Characteristics

Characteristic	No. (%) (N = 61)^a^
**Caregivers**	
Age, mean (SD), y	36.1 (5.9)
Race and ethnicity	
American Indian or Alaska Native	2 (3.3)
Asian	0
Black or African American	1 (1.6)
Hispanic, Latinx, or Spanish origin	0
Middle Eastern or North African	0
Native Hawaiian or Pacific Islander	0
White	55 (90.2)
Other	0
Prefer not to say	2 (3.3)
Gender identity	
Cisgender woman	51 (83.6)
Cisgender man	1 (1.6)
Nonbinary	1 (1.6)
Prefer not to say	7 (11.5)
Education	
<High school diploma	2 (3.3)
High school diploma or GED	11 (18.0)
Some college, no degree	16 (26.2)
Associate’s degree	11 (18.0)
College degree	8 (13.1)
Graduate degree	11 (18.0)
Employment status	
Full time	23 (37.7)
Part time	8 (13.1)
Self-employed	7 (11.5)
Unemployed	12 (19.6)
Student	1 (1.6)
Disabled	5 (8.2)
Marital status	
Single, never married	17 (27.9)
Married or cohabitating	31 (50.8)
Widow or widower	1 (1.6)
Divorced	6 (9.8)
Separated	2 (3.3)
Prefer not to say	2 (3.3)
**Households**	
Household size, mean (SD), No.	
Members	3.1 (1.2)
Children	2.2 (1.0)
Annual income, $	
0-9999	5 (8.2)
10 000-19 999	7 (11.5)
20 000-29 999	16 (26.2)
30 000-39 999	9 (14.8)
40 000-49 999	9 (14.8)
50 000-59 999	7 (11.5)
60 000-69 999	2 (3.3)
≥70 000	5 (8.2)
Public welfare use	
TANF	9 (14.8)
WIC	40 (65.6)
SNAP	32 (52.5)
School lunch or breakfast programs	50 (82.0)
Medicaid	54 (88.5)
Energy assistance	29 (47.5)
Housing assistance (eg, section 8)	10 (16.4)
Childcare financial assistance	22 (36.1)
Social Security	13 (21.3)

Abbreviations: GED, General Educational Development test; SNAP, Supplemental Nutrition Assistance Program; TANF, Temporary Assistance for Needy Families; WIC, Women, Infants, and Children Nutrition Program.

^a^ For 1 participant with missing demographic survey data, age and SNAP participation data were based on information provided in their eligibility screener. All other variables were missing for this participant. All percentages are given out of 61 total participants as the denominator, with 1 individual included in the sum as missing data where applicable.

### Missing Data and Attrition

A total of 58 participants (95.1%) were retained for the entire study duration. Overall, participants completed 27 of 30 daily surveys (90.4%), with 35 participants (57.3%) completing all surveys.

### Outcomes Among Children

Neither the iMean nor the iSD differed significantly between participants and nonparticipants in SNAP (eTable 1 in Supplement 1). Regression models found associations between household-level food insecurity and child internalizing (CBCL Internalizing Problems scale: *F* [2, 56] = 5.50; *P* = .01; *R*^2^ = 0.16) and total mental health (CBCL Total Problems scale: *F*[2,56] = 3.59; *P* = .03; *R*^2^ = 0.11) problems. Notably, the amount of instability (iSD) in household food insecurity was associated with child internalizing (CBCL Internalizing Problems scale: β = 0.40; *P* = .003) and total mental health (CBCL Total Problems scale: β = 0.34; *P* = .01) problems over and above the mean level of food insecurity (iMean) during the 1 month study period, which had no associations with these outcomes (CBCL Internalizing Problems scale: β = 0.01; *P* = .91; CBCL Total Problems Scale: β = −0.02; *P* = .89) (Table 3). Similarly, models showed associations between children’s direct experiences of food insecurity and child internalizing (CBCL Internalizing Problems scale: *F* [2,56] = 8.18; *P* <.001; *R*^2^ = 0.23) and total mental health (CBCL Total Problems scale: *F* [2,56] = 3.96; *P* = .02; *R*^2^ = 0.12) problems. Higher levels of instability in children’s daily experiences of food insecurity (iSD) were associated with more severe internalizing (CBCL Internalizing Problems scale: β = 0.49; *P* < .001) and total mental health (CBCL Total Problems scale: β = 0.35; *P* = .01) problems over and above the mean level (iMean) of food insecurity, which had no association with these outcomes (CBCL Internalizing Problems scale: β = −0.03; *P* = .83; CBCL Total Problems scale: β = 0.01; *P* = .94) (Table 3). The association between instability in food insecurity and internalizing problems was more robust at the child level than the household level. Mean monthly household- and child-level food insecurity were not independently associated with any domain of child mental health problems or academic performance (Table 3).

**Table 3.** Association of Daily Variation in Food Insecurity With Child Mental Health Problems

Estimate	Household food insecurity										Child food insecurity									
	TOT	*P* value	EXT	*P* value	INT	*P* value	ATT	*P* value	ACA	*P* value	TOT	*P* value	EXT	*P* value	INT	*P* value	ATT	*P* value	ACA	*P* value
iMean, β	−0.02	.89	0.06	.68	0.01	.91	0.09	.30	−0.03	.81	0.01	.94	0.11	.46	−0.03	.83	−0.17	.24	−0.04	.79
iSD, β	0.34	.01	0.10	.48	0.40	.003	−0.14	.51	−0.11	.45	0.35	.01	0.15	.29	0.49	<.001	0.16	.27	<0.01	.98
*R* ^2^	0.11	NA	0.02	NA	0.16	NA	0.02	NA	0.02	NA	0.12	NA	0.05	NA	0.23	NA	0.03	NA	<0.01	NA
*F* [2,56]	3.59	.03	0.48	.62	5.50	.01	0.62	.54	0.40	.67	3.96	.02	1.45	.24	8.18	<.001	0.93	.40	0.04	.96

Abbreviations: ACA, CBCL Academic Performance Scale; ATT, CBCL Attention Problems Scale; CBCL, Child Behavior Checklist; EXT, CBCL Externalizing Problems Scale; iMean, intraindividual mean; INT, CBCL Internalizing Problems Scale; iSD, intraindividual SD; NA, not applicable; TOT, CBCL Total Problems Scale.

### Outcomes Among Caregivers

Intraclass correlations for daily items ranged from 0.03 (daily food insecurity question No. 5) to 0.69 (daily food insecurity question No. 4), indicating that all items contained adequate within-person variability for multilevel modeling (eTables 2 in Supplement 1). Using multilevel confirmatory factor analysis, daily items were organized into the following constructs: household food insecurity, negative affect, effective action, impulse and attentional control, and parent-child conflict (eAppendix 3 in Supplement 1).

The final MSEM model, including covariates and time indices, produced acceptable fit to the data (χ^2^ = 1481.38; *df = *506; *P* < .001; CFI = 0.91; RMSEA = 0.04; SRMR = 0.06 at the within-person level and 0.10 at the between-person level). The Figure presents standardized coefficients for model paths. At the within-person level, daily food insecurity was positively associated with daily hunger (path *d*_4_). Daily food insecurity and hunger were associated with caregiver negative affect (paths *d*_3_ and *g*_3,_ respectively), effective action (paths *d*_1_ and *g*_1_, respectively), and attention and impulse control (paths *d*_2_ and *g*_2_, respectively), such that higher levels of food insecurity and hunger were independently associated with higher same-day reports of negative affect and lower same-day reports of perceived effectiveness and attention and impulse control. Indirect paths from food insecurity to parent-child conflict via negative affect (path *d*_3_*e*_3_; β = 0.06; *P* < .001) and attention and impulse control (path *d*_2_*e*_2_; β = 0.02; *P* = .04) were significant. Furthermore, sequential mediation paths via hunger and negative affect (*d*_4_*g*_3_*e*_3_; β = .02; *P* = .001) and hunger and attention and impulse control (*d*_4_*g*_2_*e*_2_*; *β = 0.01; *P* = .04) were also significant. The amount of time spent with children was a significant covariate for parent-child conflict (β = 0.23; *P* < .001)*_._* In addition, there were temporal associations between days since benefit transfer and negative affect (β = −0.06; *P* = .03) and between days since the start of the study and attention and impulse control (β = 0.19; *P* < .001). This within-person model explained 17% of the total variability in daily parent-child conflict (R^2^ = 0.17).

**Figure.**
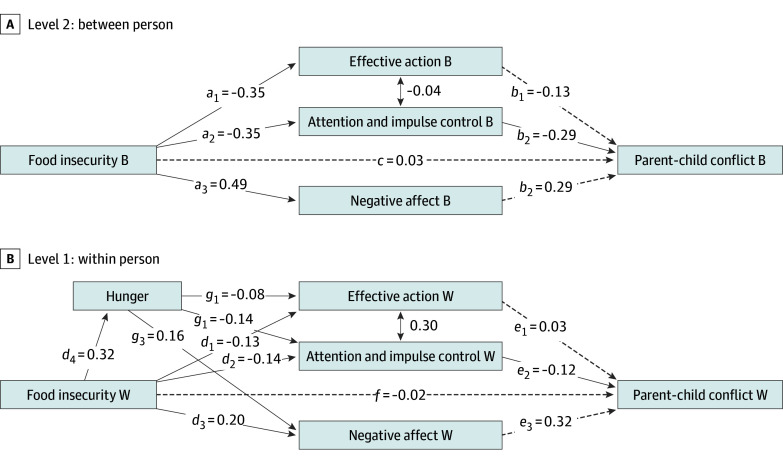
Standardized Estimates of Association Between Food Insecurity and Parent-Child Conflict B indicates between; dashed lines, significance (association); solid lines, nonsignificance (no association); W within. Estimates are shown for the multilevel sequential mediation model. The 2-way arrows indicate correlations.

At the between-person level, food insecurity was positively associated with caregiver negative affect and negatively associated with caregiver effective action and attention and impulse control. No direct or indirect associations between food insecurity and parent-child conflict were identified at the between-person level. Caregiver age, mental health symptoms, IQ, and physical health were significant covariates. Specifically, more severe caregiver mental health symptoms were associated with higher mean levels of negative affect (β = 0.37; *P* = .002) and lower mean levels of effective action (β = −0.28; *P* = .01) and attention and impulse control (β = −0.46; *P* < .001). IQ scores were positively associated with mean levels of negative affect (β = 0.24; *P* = .03) and parent-child conflict (β = 0.35; *P* = .002). Younger caregiver age was associated with higher mean levels of parent-child conflict (β = −0.37; *P* < .001), and better physical health was associated with greater effective action (β = 0.27; *P* = .02) (Figure). This between-person model explained 59% of the variability in mean parent-child conflict (*R*^2^ = 0.59).

## Discussion

This cross-sectional study expands on previous literature regarding the association between food insecurity and negative psychological outcomes among children and caregivers^14,43,44^ and provides insights into potential mechanisms that may be at play. Notably, to our knowledge, this was the first study to quantify the magnitude of day-to-day variability, in addition to the overall severity of food insecurity. We demonstrated that greater daily fluctuations in levels of household food insecurity were associated with more severe child mental health problems, especially internalizing problems, than the overall severity of household food insecurity. These findings hint at the role that household chaos may play in the development and maintenance of mental health problems among children in food-insecure households. Children benefit from routine and predictability,^45,46^ and family routines have been shown to promote resilience and self-regulation in children whose families are facing socioeconomic stress and adversity.^47,48,49^ However, for food-insecure households, the pressing need to feed one’s family often necessitates the use of various, creative strategies (eg, traveling to multiple stores for the lowest prices, skipping meals, and going to friends’ or families’ homes for free meals) that may detract from the routinization of family life and be associated with impaired caregiver mood and executive function.^50,51^

Relatedly, this study also examined caregiver-level experiences of food insecurity that may contribute to poor mental health outcomes among children. The novel use of MSEM allowed for indirect, within-person and within-household processes to be uncovered. Specifically, results showed that on days when household food insecurity was more severe, caregivers experienced higher levels of negative affect and perceived themselves as less effective and able to regulate their attention and impulses. Higher daily food insecurity was indirectly associated with parent-child conflict via caregiver daily experiences of hunger, negative affect, and attention and impulse control. While heightened negative affect has been well-documented among caregivers experiencing food insecurity^52,53,54^ and as a risk factor associated with less-effective parenting,^55,56^ the role of caregiver attention and impulse control in the association between household food insecurity and parent-child conflict is an important addition. This finding aligns well with parenting literature that has long acknowledged the critical importance of caregiver attention in the parent-child relationship and child psychological functioning.^57^ Through this lens, food insecurity is viewed as a distal stressor that distracts from the proximal task of parenting, leading to diminished caregiver capacity to appropriately monitor child behaviors, as well as the caregiver’s own responses, resulting in harsher, more reactive parenting. Crucially, caregiver attentional challenges can be associated with less effective parenting even among individuals who possess strong parenting knowledge and positive parenting attitudes.^50,57^ This is consistent with our finding that higher caregiver IQ was associated with more parent-child conflict.

These findings carry policy implications. Programs such as SNAP may increase caregiver stress by requiring more than 16 hours of weekly meal preparation to stretch benefits^58,59^ and threaten household stability as many families find themselves in a feast-famine cycle due to the inconsistent availability of resources.^60^ Our study highlights the need for hunger-alleviation programs to value and conserve the time and cognitive and emotional resources of households affected by food insecurity.

### Limitations

This study has several limitations. The data for this study were collected in the middle stages of the COVID-19 pandemic. During this time, many families faced unprecedented levels of stress but also benefitted from the expansion of federal and local assistance programs intended to alleviate pandemic-related hardships. In particular, temporary, emergency allotments of SNAP benefits were issued, which allowed all participating households to receive the maximum monthly benefit amount regardless of income,^61,62^ and federal school meal programs were expanded to make free breakfasts and lunches available to all students. As a result, data collected in this study may not represent the experiences of rural, low-income families outside of the context of the COVID-19 pandemic.

There was also very limited diversity among caregivers in our sample given that the vast majority represented White, female parents and reflect the overrepresentation of single mothers in food-insecure households.^42,63^ As such, the generalizability of these findings to other populations affected by food insecurity is unclear. There are also some limitations of daily-diary methods, including reliance on data from a single informant. We attempted to mitigate this by collecting teacher reports of child classroom functioning; however, we could not analyze these data owing to very low completion rates. Notably, many caregivers declined consent for teacher contact, which may reflect stigma related to food insecurity and the need for research institutions to build greater trust among rural, low-income communities.

## Conclusions

This cross-sectional study of household food insecurity and child mental health problems provides support for the potential role of parenting stress, demonstrating that caregiver experiences of hunger, negative affect, and attention and impulse control fully mediated daily associations between food insecurity and parent-child conflict. These findings underscore the need for programs and policies that go beyond relieving hunger by focusing on emotional, cognitive, and behavioral associations among children and families. Our research suggests that enhancing stability and routinization among food-insecure households and decreasing the cognitive burden of food insecurity on caregivers may be especially helpful.

## Notes

Supplement 1. **eAppendix 1.** Measures for covariates
eReferences.
**eAppendix 2.** Mathematical equations for intraindividual means and standard deviations **eTable 1.** Independent samples t-tests comparing iMean and iSDs by SNAP participation **eTable 2.** Unconditional means models and intraclass correlations for primary daily variables **eAppendix 3.** Fit for multilevel confirmatory factor analysis models

Supplement 2.
Data Sharing Statement
