# Supplementary material for: Food Insecurity and Rural Child and Family Functioning
Source: JAMA Netw Open. 2025 Sep 5;8(9):e2530691. doi: 10.1001/jamanetworkopen.2025.30691 (PMC12413651; doi:10.1001/jamanetworkopen.2025.30691)
Supplement: Supplement 1. — eAppendix 1. Measures for covariates eReferences. eAppendix 2. Mathematical equations for intraindividual means and standard deviations eTable 1. Independent samples t-tests comparing iMean and iSDs by SNAP participation eTable 2. Unconditional means models and intraclass correlations for primary daily variables eAppendix 3. Fit for multilevel confirmatory factor analysis models [file jamanetwopen-e2530691-s001.pdf]

Supplemental Online Content

Ametti MR, Frering HE, Huang K, Marsh K, Althoff RR. Food insecurity and rural child and family functioning. *JAMA Netw Open*. 2025;8(9):e2530691.
doi:10.1001/jamanetworkopen.2025.30691

eAppendix 1. Measures for covariates

eReferences.

eAppendix 2. Mathematical equations for intraindividual means and standard deviations

eTable 1. Independent samples t-tests comparing iMean and iSDs by SNAP participation

eTable 2. Unconditional means models and intraclass correlations for primary daily variables

eAppendix 3. Fit for multilevel confirmatory factor analysis models

This supplemental material has been provided by the authors to give readers additional information about their work.

© 2025 Ametti MR et al. *JAMA Network Open*.

eAppendix 1: Measures for covariates. Caregiver age and gender were derived from a self-report demographics questionnaire. Caregivers' IQ was approximated using the Raven's Advanced Progressive Matrices administered remotely via Pearson Assessment's Q-Global platform[1]. To reduce participant burden, the 24-item short form version was used, which has been shown to correlate highly (r=0.93) with the full 48-item version of the assessment. In this task, participants were presented with a series of increasingly difficult geometric patterns, each which contained a missing element that they were asked to fill in by selecting among either six or eight multiple choice options. Based on the participant's performance and age, an IQ score between 70 and 130 was generated, with scores between 85 and 115 representing average cognitive abilities. Caregivers' overall mental health was assessed using the Adult Self Report[2], a self-report inventory on which caregivers rated the extent to which they have experienced 126 psychological problems during the past six months on a three-point scale (0 = *not true,* 1 = *somewhat or sometimes true*, 2 = *very true or often true*). Items form empirically validated subscales that are normed by age, gender, and culture. *T*-scores (*M*=50, *SD*=10) on the Total Problems subscale were used as a measure of caregivers' overall level of mental health concerns. This scale has excellent internal validity (Cronbach's alpha = 0.97) and reliability (test-rest r = 0.94). Finally, caregivers' overall physical health was assessed using the Physical Functioning subscale of the Short Form Health Survey[3] (SFHS) was used to measure caregivers' general physical health. The SFHS is an empirically valid and disease non-specific, 36-item self-report measure that assesses physical functioning, mobility, pain, and associated interference with typical activities. Items are averaged to yield a score between 0 and 100, with higher scores representing better health. The Physical Functioning subscale of the Short Form Health Survey has been demonstrated to have relatively little overlap with mental health symptoms and is sensitive to the presence of medical conditions[4].

© 2025 Ametti MR et al. *JAMA Network Open*.

eReferences

1. Arthur, W., & Day, D. V. Development of a short form for the Raven Advanced Progressive Matrices Test. Educational and Psychological Measurement, 1994; 54(2), 394–403.

2. Achenbach TM, Rescorla LA. Manual for the ASEBA Adult Forms & Profiles. Burlington, VT: University of Vermont, Research Center for Children, Youth, & Families; 2003.

3. Ware JE Jr, Sherbourne CD. The MOS 36-item short-form health survey (SF-36). I. Conceptual framework and item selection. Med Care. 1992 Jun;30(6):473-83. PMID: 1593914.

4. McHorney CA, Ware JE Jr, Raczek AE. The MOS 36-Item Short-Form Health Survey (SF-36): II. Psychometric and clinical tests of validity in measuring physical and mental health constructs. Med Care. 1993 Mar;31(3):247-63. doi: 10.1097/00005650-199303000-00006. PMID: 8450681.

© 2025 Ametti MR et al. *JAMA Network Open*.

eAppendix 2: Mathematical equations for intraindividual means and standard deviations.

i*Mean*	i*SD*
$M_{\mathrm{FI}j} = \frac{1}{T_j} \sum_{t=1}^{T_j} \mathrm{FI}_{tj}$	$SD_{\mathrm{FI}j} = \sqrt{\frac{\sum_{t=1}^{T_j} (\mathrm{FI}_{tj} - M_{\mathrm{FI}})^2}{T_j - 1}}$

FI = food insecurity; *T* = total number of time points; *t* = specific time point; *j* =specific person

© 2025 Ametti MR et al. *JAMA Network Open*.

eTable 1. Independent Samples T-Tests Comparing iMean and iSDs by SNAP Participation

		M		***t (df)***	**95% CI**	***p***
		SNAP (n = 32)	*Non-SNAP* (n = 29)			
iMean	Household Food Security	1.73	1.51	-0.50 (50.35)	[-0.97 – 0.53]	.56
	Child Food Security	0.50	0.46	-0.25 (49.69)	[-0.31- 0.24]	.80
iSD	Household Food Security	0.94	0.87	-0.65 (55.26)	[-0.30 – 0.15]	.52
	Child Food Security	0.31	0.27	-0.81 (55.40)	[-0.14 – 0.05]	.42

© 2025 Ametti MR et al. *JAMA Network Open*.

eTable 2. Unconditional means models and intraclass correlations for primary daily variables

Daily Item	Item Scale	*M*	*SE*	ICC
**Food Insecurity**				
1. worried about food running out	0 – 1	0.30	0.04	0.38
2. unable to afford balanced meals	0 – 2	0.63	0.06	0.55
3. ate less than you felt you should	0 – 1	0.22	0.04	0.47
4. unable to feed children balanced meals	0 – 2	0.48	0.07	0.69
5. children ate less than you felt they should	0 – 1	0.01	0.00	0.03
**Negative Affect**				
1.depressed	1 – 5	1.99	0.11	0.51
2. angry	1 – 5	1.51	0.06	0.30
3. anxious	1 – 5	2.11	0.10	0.50
4. irritable	1 – 5	1.84	0.08	0.38
5. ashamed	1 – 5	1.36	0.64	0.39
**Executive Function**				
1. able to get started on planned activities	1 – 5	2.93	0.09	0.31
2. follow through on plans	1 – 5	3.05	0.09	0.37
3. do things that require time and effort	1 – 5	3.00	0.09	0.40
4. keep your mind on what you were doing	1 – 5	2.98	0.09	0.44
5. make up your mind about things	1 – 5	3.06	0.09	0.48
6. make mistakes †	1 – 5	4.07	0.08	0.42
7. act on impulse †	1 – 5	4.36	0.08	0.46
8. forget to do things †	1 – 5	4.17	0.08	0.47
**Parenting**				
1. showed love and affection to child	1 – 4	3.65	0.06	0.47
2. felt angry w/ child	1 – 4	1.33	0.04	0.25
3. had fun or laughed w/ child	1 – 4	3.33	0.07	0.41
4. good deal of conflict w/ child	1 – 4	1.29	0.03	0.17
5. was with child but not interacting	1 – 4	1.59	0.06	0.34
6. felt disappointed by child	1 – 4	1.07	0.02	0.22
7. too tired to interact w/ child	1 – 4	1.48	0.06	0.34
8. yelled at child	1 – 4	1.32	0.04	0.29
**Food-Based Coping Strategies**				
1. food bank	0 – 1	0.05	0.01	0.10
2. borrow money	0 – 1	0.04	0.01	0.07
3. other food-based coping strategy	0 – 1	0.10	0.02	0.27
4. any coping strategy	0 – 3	0.17	0.02	0.19
**Other**				
1.hungry	1 – 5	1.80	0.08	0.39
2. time spent with children	0 – 4	3.27	0.07	0.28

Note. † indicates reversed-coded items

© 2025 Ametti MR et al. *JAMA Network Open*.

eAppendix 3: Fit for multilevel confirmatory factor analysis models

Household Food Insecurity. Figure S3a shows the final multi-level CFA model of household food insecurity. The overall fit was good (χ^2 [*df*=4] = 37.23, *p* <.001; CFI = 0.97, RMSEA = 0.07; and $SRMR_{Within}/SRMR_{Between}$ = 0.03/0.06). In addition, partially saturated models showed no evidence of misfit localized at either level of the model and the level-specific fit was excellent for the within-person level model (χ^2 [*df* = 2] = 8.33, *p* < .02; CFI = 0.98; RMSEA = 0.04; and $SRMR_{Within}/SRMR_{Between}$ = 0.03/0.001) and good for the between-person level model (χ^2 [*df* = 2] =20.42, *p* < .001; CFI = 0.98; RMSEA = 0.08; and $SRMR_{Within}/SRMR_{Between}$= <0.001/0.06).

All four daily items related to food insecurity had moderate to strong factor loadings, ranging from 0.39 to 0.77 at the within-person level and from 0.65 to 0.97 at the between-person level. Scores on the latent factor measured within-person changes in household food insecurity across days with moderate reliability (ω = 0.68) and between-person differences in with high reliability (ω = 0.93).

Figure S3a. *Final multilevel CFA model of household food insecurity.*

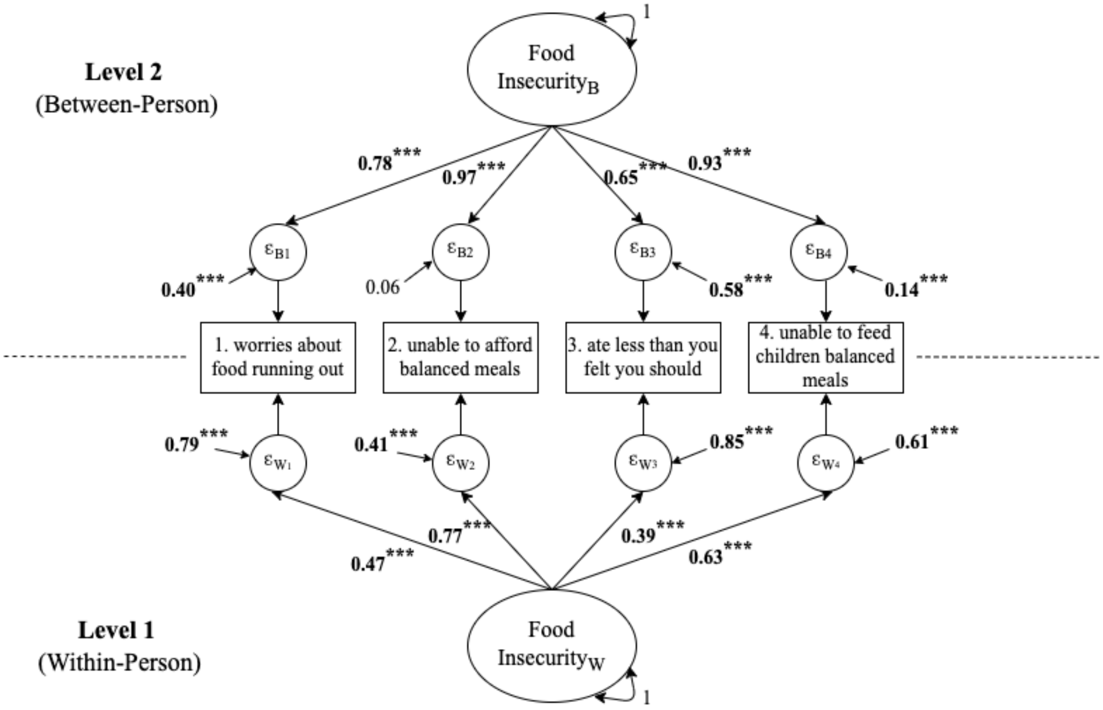

Note. * *p* < .05, ** *p* <.01, ****p* <.001; ε = *p*-variate residual (specific error + measurement error)

© 2025 Ametti MR et al. *JAMA Network Open*.

Caregiver Negative Affect. Figure S3b shows the final, one-factor multi-level CFA of caregiver negative affect, which had excellent overall model fit (χ^2 [*df*=8] = 38.92, *p*<.001; CFI = 0.98; RMSEA = 0.05; $SRMR_{Within}/SRMR_{Between}$ = 0.02/0.03). Partially saturated models confirmed that fit was consistent for both the within-person (χ^2 [*df*=4] = 14.83, *p*<.005; CFI = 0.99; RMSEA=0.04; $SRMR_{Within}/SRMR_{Between}$ = 0.02/ <0.01) and the between-person level (χ^2 [*df*=4] = 7.76, *p* =.10; CFI = 1.00; RMSEA = 0.02; $SRMR_{Within}/SRMR_{Between}$ = <0.01/0.03) models.

All daily items were moderate to strong indicators of the latent construct as evidenced by standardized factor loadings ranging from 0.48 to 0.69 at the within-person level and from 0.57 to 0.93 at the between-person level. Omega coefficients were 0.72 and 0.69 for the within- and between-person levels, respectively, indicating that the latent factor measured both within-person changes across days and between-person differences in negative affect with moderate reliability.

Figure S3b. *Final multilevel CFA model of caregiver negative affect.*

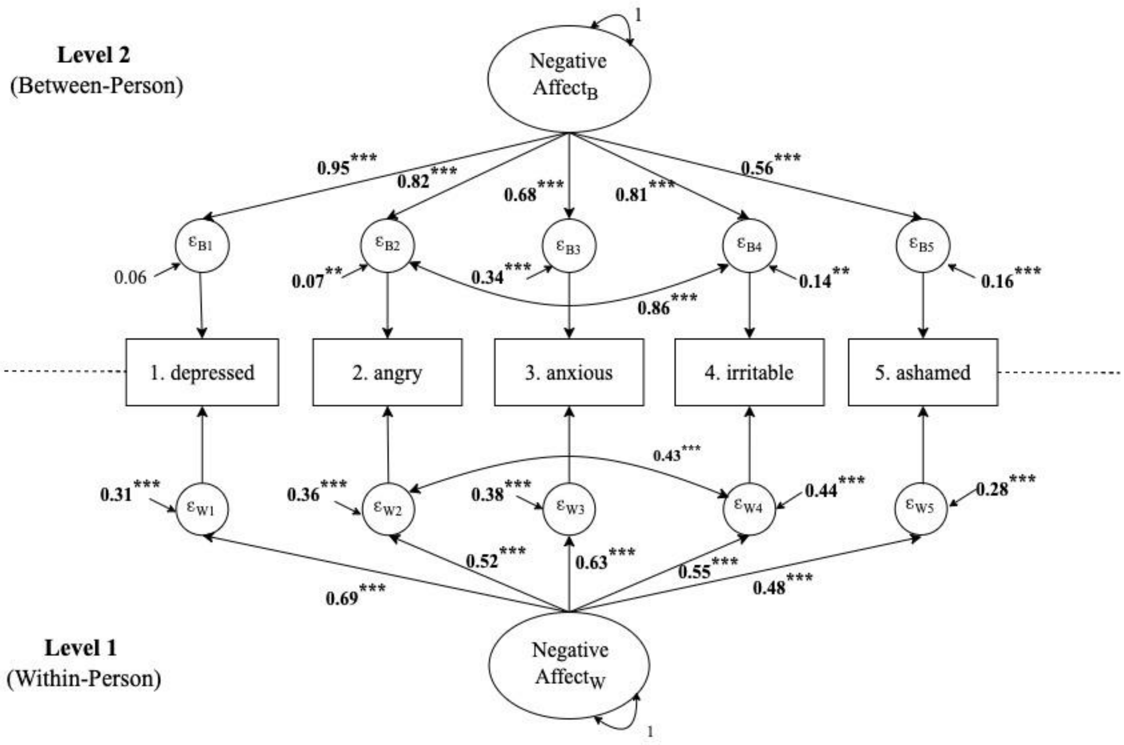

Note. * *p* < .05, ** *p* <.01, ****p* <.001; ε = *p*-variate residual (specific error + measurement error)

Caregiver Executive Functioning. The final, two-factor multi-level CFA model of caregiver executive functioning shown in Figure S3c demonstrated excellent overall fit (χ^2 [*df*= 38] =234.88, *p*<.001; CFI = 0.95; RMSEA = 0.06; $SRMR_{Within/}SRMR_{Between}$ = 0.03/0.05). In partially saturated models, this was consistent for both the within-person (χ^2 [*df*= 19] =130.09, *p*<.001; CFI = 0.97; RMSEA = 0.06; $SRMR_{Within/}SRMR_{Between}$ = 0.03/<0.01) and between-person (χ^2 [*df*= 19] = 68.37, *p*<.001; CFI = 0.99; RMSEA = 0.04; $SRMR_{Within/}SRMR_{Between}$ = <0.01/0.05) specific models.

The first factor, labeled as *Effective Action,* consisted of five items (i.e., "able to get started on planned activities," "following through on plans," "doing things that require time and effort," "keeping your mind on what you were doing," and "making up your mind about things"). All items were strong indicators of the latent factor as evidenced by standardized factor loadings ranging from 0.72 to 0.85 at the within-person level and from 0.98 to 1.00 at the between-person level. The remaining three items (i.e., "makes mistakes," "acts on impulse," and "forgets to do things") all loaded positively onto a second factor, labeled *Attention/Impulse Control*. These items were moderate-to-strong indicators of the latent construct, ranging from 0.37 to 0.69 at the within-person level and from 0.62 to 0.98 at the between-person level. At both levels of analysis, the two factors were weakly, positively correlated, suggesting good differentiation between the latent constructs. The *effective action* factor was highly reliable at both levels of analysis with a between-level ω = 0.99 and within-level ω =0.89, whereas the *attention/impulse control* factor was highly reliable at the between-person level (ω = 0.91) and moderately reliable at the within-person level (ω = 0.54).

Figure S3c. Final multilevel CFA model of caregiver executive function

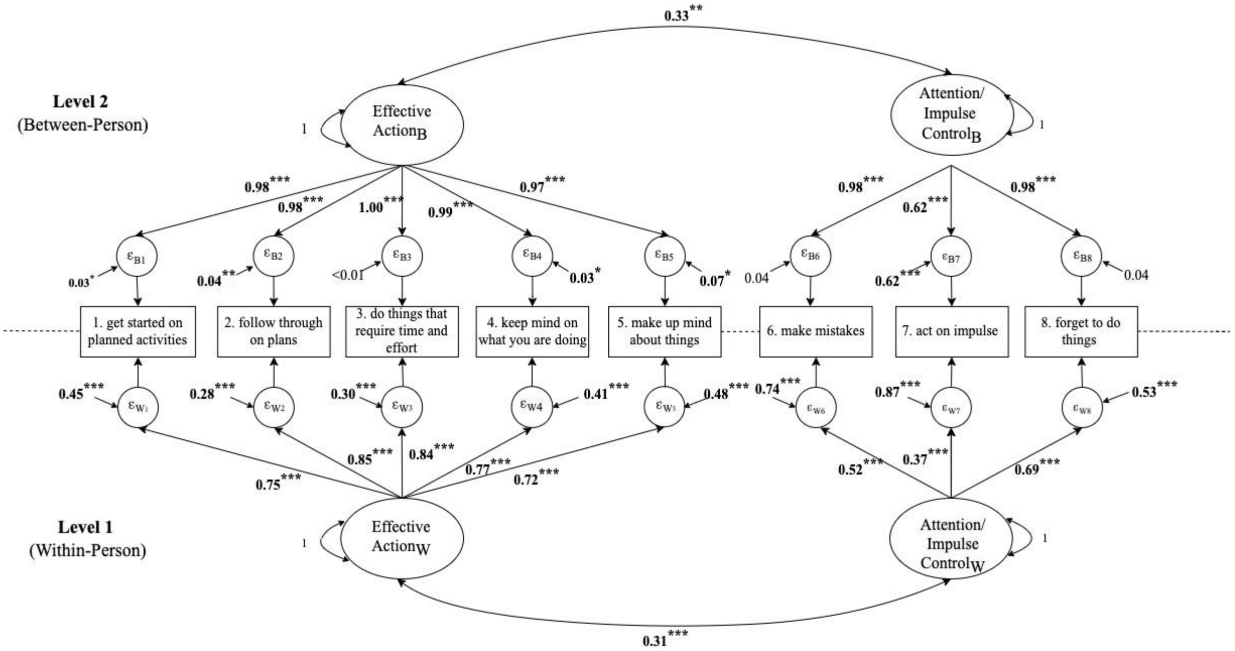

© 2025 Ametti MR et al. *JAMA Network Open*.

Note. * p < .05, ** p <.01, ***p <.001; ε = p-variate residual (specific error + measurement error)

Parenting-Child Conflict. Figure S3d shows the final one-factor multi-level CFA model of *parent-child conflict,* which fit the data well (χ^2 [*df*= 4] = 26.62, p<.001; CFI = 0.98; RMSEA = 0.06; $SRMR_{Within}/SRMR_{Between}$ = 0.02/0.05). Level-specific fit indices did not provide any evidence of localized misfit at either the within-person (χ^2 [*df*= 2] = 18.49, p<.001; CFI = 0.99; RMSEA = 0.07; $SRMR_{Within}/SRMR_{Between}$ = 0.02/<0.01) or between-person levels (χ^2 [*df*= 2] = 8.38, p<.02; CFI = 1.00; RMSEA = 0.05; $SRMR_{Within}/SRMR_{Between}$ = <0.01/0.05).

Standardized factor loadings ranged from 0.45 to 0.70 at the within-person level and from 0.47 to 1.00 at the between-person level, suggesting that items were moderate-to-strong indicators of the latent factor. The factor measured between-person differences with high reliability (ω = 0.94), whereas changes across days were measured with moderate reliability (ω = 0.74).

Figure S3d. Final multilevel CFA model of parent-child conflict.

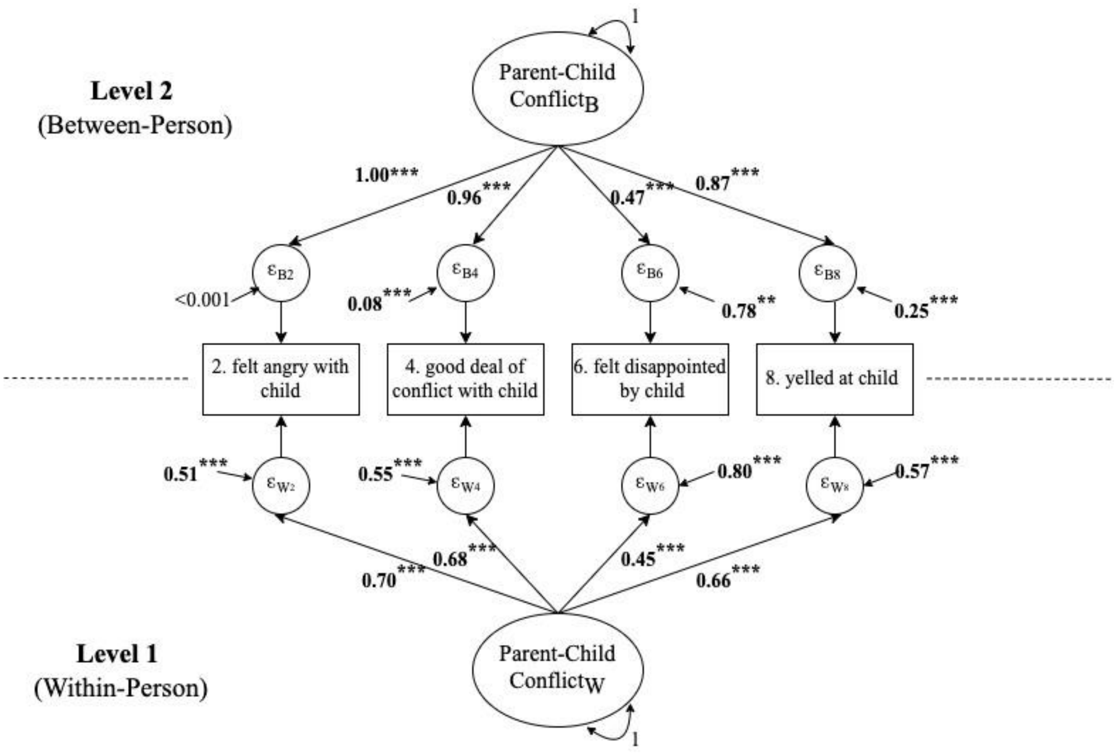

Note. * p < .05, ** p <.01, *** p <.001; ε = p-variate residual (specific error + measurement error)

© 2025 Ametti MR et al. *JAMA Network Open*.
