# Supplementary material for: Food Insecurity and Rural Child and Family Functioning
Source: JAMA Netw Open. 2025 Sep 5;8(9):e2530691. doi: 10.1001/jamanetworkopen.2025.30691 (PMC12413651; doi:10.1001/jamanetworkopen.2025.30691)
Supplement: Supplement 2. — Data Sharing Statement [file jamanetwopen-e2530691-s002.pdf]

Data Sharing Statement

Ametti. Food Insecurity and Rural Child and Family Functioning. *JAMA Netw Open*. Published online September 5, 2025. doi:10.1001/jamanetworkopen.2025.30691

Data
Data available: Yes
Data types: Deidentified participant data
How to access data: De-identified data and will be available by request to Principal Investigator with data use agreement.
When available: With publication

Supporting Documents
Document types: None

Additional Information
Who can access the data: Data will be made available to any researcher with IRB approval and data use agreement.
Types of analyses: for any IRB approved secondary analysis
Mechanisms of data availability: with a signed data use agreement
Any additional restrictions: n/a
